# DHT-Induced lncRNA AC092718.4 Promotes Prostate Cancer Cell Proliferation via ceRNA Mechanism

**DOI:** 10.3390/genes17050538

**Published:** 2026-05-01

**Authors:** Lian Jin, Shan Feng, Wei-Jie Sun, Jun Ouyang, Feng Liu, Bai-Cheng Lu, Ya-Ping Zhang, Hui Zhao

**Affiliations:** 1 School of Life Sciences, Yunnan University, Kunming 650500, China; jinlian_2016@163.com (L.J.); pinggaipingsai1@foxmail.com (S.F.); oouyyang@foxmail.com (J.O.); liu973991663@163.com (F.L.); lu_baicheng@163.com (B.-C.L.); 2 Bio-X Center for Interdisciplinary Innovation, Yunnan University, Kunming 650500, China; sunweijie@ynu.edu.cn; 3 State Key Laboratory of Genetic Evolution & Animal Models, Kunming Institute of Zoology, Chinese Academy of Sciences, Kunming 650201, China; 4 Center for Excellence in Animal Evolution and Genetics, Chinese Academy of Sciences, Kunming 650223, China

**Keywords:** AC092718.4, androgen, lncRNAs, prostate cancer, ceRNA

## Abstract

**Background/Objectives**: The androgen receptor (AR)-driven transcriptional program plays a pivotal role in the development and progression of prostate cancer. The binding of androgen dihydrotestosterone (DHT) to AR initiates transcriptional activation, thereby altering the transcriptional landscape. DHT-induced long non-coding RNAs (lncRNAs) have been recognized as crucial players in prostate cancer pathogenesis. This study aims to identify and explore the important role of such lncRNAs in prostate cancer. **Methods**: This study first analyzed transcriptome data from an androgen-dependent cell line, LNCaP, treated with different DHT concentrations and found a batch of lncRNAs exhibiting DHT concentration dependence. TCGA data suggested a correlation between the DHT-induced lncRNA and prostate cancer. Finally, a series of in vivo and in vitro experiments confirmed the effect and mechanism of lncRNA in prostate cancer. **Results**: AC092718.4 was highly expressed in AR-positive prostate cancer cell lines and tissues, and its expression in patients with Gleason scores 6–9 was significantly higher than in a normal control group. Notably, the expression level of AC092718.4 was upregulated in a concentration-dependent manner with DHT. In vitro experiments revealed that overexpression of AC092718.4 promoted cell proliferation and inhibited cell apoptosis. Conversely, knockdown of AC092718.4 suppressed tumorigenesis in vivo. Furthermore, our investigation into the pathogenetic mechanism demonstrated that AC092718.4 could act as an miRNA sponge for miR-138-5p, attenuating its inhibitory effect on downstream oncogenes, such as *FERMT2*, *RHOC*, and *HIF1A*. These AC092718.4/miR-138-5p/mRNA axes, in turn, facilitated the progression of prostate cancer. **Conclusions**: For the first time, we demonstrate that AC092718.4 may function as an oncogenic factor in prostate cancer. The AC0927.8.4/miR-138-5p/mRNA axes potentially offer promising diagnostic and therapeutic targets for prostate cancer.

## 1. Introduction

Prostate cancer ranks as the second most prevalent cancer globally and stands as the fifth leading cause of cancer-related deaths among men worldwide [1], imposing a substantial burden on both individuals and society. The androgen receptor (AR) is a ligand-dependent transcription factor. AR signaling plays an important role not only in maintaining normal prostate function but also in driving the progression of prostate cancer [2,3]. Testosterone, the most abundant androgen, serves as the predominant ligand for AR under physiological conditions [2,4]. It can be converted into the more potent derivative dihydrotestosterone (DHT) by 5α-reductase [4]. The binding of testosterone or DHT to AR triggers AR activation and subsequent nuclear translocation. Subsequently, AR dimers bind to the androgen response elements (AREs) of target genes, thereby initiating transcriptional reprogramming [2,5,6].

Approximately 1.5% to 4.3% of the transcriptome in prostate cancer LNCaP cells is regulated directly or indirectly by androgens [7]. Our previous study also confirmed that DHT induces extensive alternative polyadenylation events in AR-positive (AR^+^) prostate cancer cells [8]. The fluctuation underscores the profound impact of androgens on gene expression and highlights the importance of the AR signaling pathway and its downstream target genes in the pathogenesis of prostate cancer. Consequently, based on the mechanism of reducing androgen levels and AR signaling activity to decelerate the progression of prostate cancer, surgical or medical castration, namely androgen deprivation therapy (ADT), has long been the mainstream therapeutic approach. However, the majority of patients gradually develop resistance and eventually progress to castration-resistant prostate cancer (CRPC) [2,4]. Therefore, employing pharmacological and genetic interventions to reverse therapy resistance is emerging as a promising option [9].

In prostate cancer, androgens not only affect coding RNA but also extensively affect non-coding RNA, including long non-coding RNAs (lncRNAs). LncRNAs, defined as non-coding transcripts longer than 200 nucleotides [10], exert regulatory influences on gene expression at multiple levels, including chromatin, transcriptional, and post-transcriptional levels [11]. A growing number of studies have demonstrated that lncRNAs play crucial roles in cell proliferation, migration, invasion, angiogenesis, drug resistance, and apoptosis. The expression levels of numerous lncRNAs fluctuate in prostate cancer, endowing them with the potential to serve as diagnostic markers. For instance, the prostate-specific lncRNA prostate cancer antigen 3 (PCA3) is overexpressed in the majority of prostate cancer tumors and has been approved by the Food and Drug Administration (FDA) as a diagnostic marker for prostate cancer [12]. The activation of AR signaling is closely implicated in the dysregulation of numerous lncRNAs in prostate cancer. An increasing number of studies have identified lncRNAs that are highly regulated in prostate cancer in an androgen-dependent manner, such as PART1, CBLL1-AS1, DUBR, SOC2-AS1, PVT1, SNHG5, POTEF-AS1, FAM83H-AS1, and CTBP1-AS [13,14,15,16]. These androgen-related lncRNAs provide more clues for the study of the pathogenic mechanism of prostate cancer and ADT.

One of the most fascinating functions of lncRNAs is their ability to function as competing endogenous RNAs (ceRNAs) or miRNA sponges. They compete with endogenous mRNAs for miRNAs via miRNA recognition elements (MREs), thereby alleviating miRNA-mediated repression of downstream mRNAs [17,18,19]. An increasing number of studies have shown that the interaction among these three molecules forms an lncRNA/miRNA/mRNA axis, which plays a crucial role in prostate cancer. Furthermore, given the bifunctional roles of lncRNAs or miRNAs, which can act as both tumor suppressors and oncogenes, and the crosstalk among molecules or axes that forms a more extensive and complex ceRNA network, targeting these interactions is undoubtedly likely to yield better therapeutic outcomes with fewer off-target effects [19]. Consequently, the ceRNA networks consisting of lncRNA/miRNA/mRNA axes represent promising therapeutic targets in prostate cancer. Specifically, these androgen-related lncRNAs may provide new options for the treatment of intractable CRPC.

Recent studies uncovered the pathogenic mechanisms by which DHT-induced lncRNAs act as miRNA sponges in prostate cancer. The androgen response-related lncRNA PVT1 functions as a sponge for miRNA-186-5p to upregulate endothelial–mesenchymal transition-related Twist1, thereby promoting prostate cancer invasion and metastasis [20]. DHT-induced lncRNA PART1, together with the other 4 lncRNAs, 5 miRNAs, and 17 mRNAs, constitutes the ceRNA network of prostate cancer and serves as one of the hub genes [21]. These interactions underscore the significance of the lncRNAs/miRNA/mRNA axis in prostate cancer progression. Meanwhile, the ceRNA axis has naturally emerged as a promising target for overcoming drug resistance.

To elucidate the role of androgen-induced lncRNAs in prostate cancer, we screened for DHT-induced lncRNAs using transcriptome analysis in the LNCaP cell line, which is an androgen-dependent prostate cell line [22]. Among the candidate differentially expressed lncRNAs (DElncRNAs), AC092718.4 was identified as a novel DHT-induced lncRNA. It is highly expressed in many types of cancers, particularly in prostate cancer, yet its functional role remains to be elucidated. In the present study, we explored the biological function of AC092718.4 and propose that it is a novel key gene in prostate cancer. Additionally, we investigated the potential mechanism that contributes to disease progression via the ceRNA axis.

## 2. Materials and Methods

### 2.1. Cell Culture and Androgen Treatments

The selection criteria for the prostate-related cell lines used in this study were based on previous research [23]. The cell lines used in this study were purchased from Kunming Cell Bank, Kunming Institute of Zoology, Chinese Academy of Sciences. Human prostate cancer cell lines (LNCaP, 22RV1, and DU145) were cultured in RPMI1640 medium (Gibco, Grand Island, NY, USA) containing 10% Charcoal-Stripped FBS (CS-FBS) (Biological Industries, Kibbutz Beit Haemek, Israel) and 1% penicillin–streptomycin (BasalMedia, Shanghai, China). The human prostate normal cell line RWPE-1 and the prostate cancer cell line PC-3 were cultured in Dulbecco’s Modified Eagle Medium (Gibco, Grand Island, NY, USA), also supplemented with 10% CS-FBS and 1% penicillin–streptomycin. All cells were cultured in an incubator at 37 °C and 5% CO_2_. The method of using 1, 10, 100, and 1000 nM DHT (Sigma-Aldrich, St. Louis, MO, USA)) to treat LNCaP cells has been described in previous studies [8]. In AR-dependent experiments, LNCaP cells were treated with 10 nM of DHT and 10 mM of the second-generation androgen antagonist enzalutamide (Enz; MedChem Express, Monmouth Junction, NJ, USA) for 48 h; cells treated with dimethyl sulfoxide (DMSO; Solarbio, Beijing, China) served as the control group. All experiments treated with DHT, Enz, and DMSO were performed in triplicate.

### 2.2. Transcriptome Sequencing Analysis

Sequencing data used in this study were obtained from our previously published data (Sequence Read Archive (SRA) database BioProject accession number: PRJNA566256) [8]. Following quality filtering, RNA-seq data were aligned to the human reference genome (GRCh38.p13) using HISAT2 [24], and subsequent transcript assembly was performed with StringTie [25]. Gene expression quantification was conducted via featureCounts [26], and differential expression analysis was conducted using DESeq2 to identify differentially expressed genes (DEGs) across distinct DHT concentrations [27]. The fold change in gene expression induced by DHT was calculated as the expression ratio of experimental groups (1 nM, 10 nM, 100 nM, and 1000 nM DHT) relative to the control group (0 nM DHT). For DElncRNAs analysis, considering that lncRNAs exhibit lower abundance and greater natural expression variation compared with mRNAs [28,29], according to a previous study [30], log_2_FC ≥ 1 and false discovery rate (FDR) < 0.05 were set as thresholds. For differentially expressed mRNAs (DEmRNAs) analysis, in view of the subtle and mild transcriptional regulation in ceRNAs, strict thresholds may filter out weakly regulated but functionally critical genes, based on previous studies [8,31], log_2_FC ≥ 0.27 and FDR < 0.05 were set as thresholds.

### 2.3. Prediction of the Characteristics of lncRNA AC092718.4

The expression profile and Gleason Score of AC092718.4 were downloaded from the UALCAN database (https://ualcan.path.uab.edu/, accessed on 19 April 2025) [32]. The protein-coding potential of lncRNA AC092718.4 was analyzed using the LncRBase database (http://dibresources.jcbose.ac.in/zhumur/lncrbase2/start2.php, accessed on 17 April 2025) [33], while its functions and cancer hallmarks were analyzed via the LncACTdb database (http://bio-bigdata.hrbmu.edu.cn/LncACTdb/, accessed on 17 April 2025) [34]. Prediction of the ceRNA network was conducted by integrating insights from multiple specialized resources, including miRDB (https://mirdb.org/, accessed on 7 July 2024) [35,36], LncRNASNP2 (https://guolab.wchscu.cn/lncRNASNP/, accessed on 20 April 2025) [37], LncBook (https://ngdc.cncb.ac.cn/lncbook/, accessed on 20 April 2025) [38], and the NPInter database (http://bigdata.ibp.ac.cn/npinter5/, accessed on 21 April 2025) [39]. Identification of downstream target genes of miRNAs was achieved by cross-referencing predictions from miRDB (accessed on 21 February 2025), TargetScan (https://www.targetscan.org/vert_80/, accessed on 28 February 2025) [40], ENCORI/StarBase (https://rnasysu.com/encori/, accessed on 17 April 2025) [41], and the miRTarBase database (https://miRTarBase.cuhk.edu.cn/, accessed on 18 April 2025) [42], with robustness ensured by leveraging complementary data from these established platforms.

All samples mentioned in this section were derived from public databases and passed the official quality control and qualification inspection of the databases. Only samples with complete clinical information and qualified sequencing data were included in the final analysis. The statistical analysis methods for these data have already been disclosed in the corresponding literature. These data are directly cited in this article.

### 2.4. RNA Extraction and Reverse-Transcription Quantitative PCR (RT-qPCR)

Total RNA was isolated using the RNeasy^®^Plus mini Kit (QIAGEN, Dusseldorf, Germany) according to the manufacturer’s protocol. Reverse transcription of total RNA was carried out using the PrimeScript^TM^ RT-PCR Kit (Takara, Tokyo, Japan), while reverse transcription of miRNAs was carried out with the miRNA 1st Strand cDNA Synthesis Kit (Vazyme, Nanjing, China). qPCR was conducted by using TB Green^®^ Premix Ex Taq™ II (Tli RNaseH Plus) (Takara, Tokyo, Japan). For lncRNA and mRNA quantification, *ACTB* was used as the endogenous control. For miRNA quantification, U6 was set as the endogenous control. All primer sequences used in this study are listed in Supplementary Table S1.

### 2.5. Fluorescence In Situ Hybridization (FISH) Assay

LNCaP cells were first fixed and permeabilized, after which denatured digoxin-labeled target-specific probes—along with 18S rRNA as a control—were hybridized to the cells and incubated overnight at 42 °C. Subsequently, the samples were incubated with an HRP-conjugated secondary antibody and tyramide-labeled using the SuperBoost™ TSA kit (Thermo Fisher Scientific, Waltham, MA, USA). Next, counterstaining with 4′,6-diamidino-2-phenylindole (DAPI) (Solarbio, Beijing, China) was performed to visualize the nuclei. Finally, the signals were visualized using a fluorescence microscope (Nikon, Tokyo, Japan). The sequences of the probes used are listed in Supplementary Table S2.

### 2.6. Plasmids

A fragment of wild-type AC092718.4 was synthesized and subcloned into pCDH-CMV-MCS-EF1-GFP+Puro by Tiangen (Beijing, China). For the construction of the lentiviral vector, shRNA constructs targeting AC092718.4 were designed (Supplementary Table S3) and inserted into the lentiviral vector pSIH1-H1-Puro (obtained from Dr. J.S.). For lentiviral production, HEK293T cells were co-transfected with the indicated shRNA constructs, pMD2G (envelope plasmid, obtained from Dr. J.S.), and psPAX2 (packaging plasmid, obtained from Dr. J.S.). Using full-length pCDH-CMV-MCS-EF1-GFP+Puro-AC092718.4 as the template, wild-type AC092718.4 was subcloned into the psiCHECK-2 vector using the homologous recombination CloneExpress^®^ II One Step Cloning Kit (Vazyme, Nanjing, China). Subsequently, the binding site mutation was achieved using the Mut Express^®^ II Fast Mutagenesis Kit V2 (Vazyme, Nanjing, China). The primers used in homologous recombination and point mutation are listed in Supplementary Table S1.

### 2.7. Transient Transfection

Cells were seeded into 6-well plates, and transfection was carried out when the cells reached 50–70% confluence. For overexpression, endotoxin-free plasmids were transfected using Lipofectamine™ 3000 transfection reagent (Invitrogen, Carlsbad, CA, USA). For knockdown, siRNAs were synthesized via GenePharma (Suzhou, China) and transfected using siRNA-MATE transfection reagent (GenePharma, Suzhou, China). All transfections were performed in accordance with the manufacturer’s protocols. Each transfection treatment group consisted of 3 biological replicates. The sequences of the siRNAs used in this study are listed in Supplementary Table S3.

### 2.8. Cell Counting Kit-8 (CCK-8) Assay

Cells were seeded into 96-well plates and subjected to transfection upon reaching the appropriate confluence. At time points of 0, 24, 48, 72, and 96 h post-transfection, 10 μL of SuperKine™ Maximum Sensitivity Cell Counting Kit-8 (CCK-8) (Abbkine, Wuhan, China) solution was added to each well of cells. Subsequently, the cells were incubated at 37 °C for 1 h, after which absorbance at a wavelength of 450 nm was measured using a microplate reader (BioTek, Winooski, VT, USA).

### 2.9. Cell Cycle Flow Cytometry

LNCaP cells were seeded into 6-well plates and subjected to transfection at the appropriate confluence. At 48 h post-transfection, the cells were trypsinized, collected into 1.5 mL microcentrifuge tubes, and rinsed with PBS. Following supernatant removal, cell pellets were resuspended in 300 μL of PBS. Subsequently, 700 μL of absolute ethanol was added dropwise, and the cells were fixed at −20 °C overnight. After centrifugation and discarding the supernatant, the cells were resuspended in 200 μL of PI staining solution (Thermo Fisher Scientific, Waltham, MA, USA) and incubated in the dark at room temperature for 30 min. Finally, the stained cells were filtered through a nylon mesh and analyzed using a flow cytometer (BD Biosciences, San Jose, CA, USA).

### 2.10. Cell Apoptosis Flow Cytometry

LNCaP and 22RV1 cells were seeded into 6-well plates and subjected to transfection at the appropriate confluence. At 48 h post-transfection, the cells were trypsinized and subsequently rinsed with 1 × binding buffer. Following supernatant removal, the cell pellets were gently resuspended and diluted to a concentration of 1 × 10^6^ cells/mL. Due to the presence of a GFP tag on the overexpression vector, Annexin V-YSFluor^TM^ 647/PI Apoptosis Detection Kit (Yeasen, Shanghai, China) was used to detect apoptosis for the overexpression of AC092718.4. Annexin V-FITC (Thermo Fisher Scientific, Waltham, MA, USA) was used to detect apoptosis for the knockdown of AC092718.4. Cell apoptosis detection was monitored according to the manufacturer’s instructions. The stained cell suspensions were filtered through a nylon mesh and analyzed using a flow cytometer.

### 2.11. Xenograft Tumor Assay

Six-week-old male BALB/c nude mice were obtained from the Laboratory Animal Center of Yunnan University. 22RV1 cells were infected with sh-AC092718.4 or sh-NC lentivirus (negative control). Stable cell clones were subsequently selected using puromycin (InvivoGen, San Diego, CA, USA). For in vivo tumorigenesis assay, 4 × 10^6^ stably transfected 22RV1 cells were subcutaneously injected into the axillary region of each nude mouse (*n* = 6 per group). The body weight and tumor diameters of the mice were measured every two days, and tumor volumes were calculated using the formula: volume = 1/2 × length × width^2^. Tumor growth was continuously monitored until the maximum tumor diameters reached no more than 20 mm, at which point the mice were humanely euthanized and the tumors were excised. Tumor weights were immediately measured, and macroscopic images of the tumors were captured. Finally, an immunohistochemistry (IHC) assay was performed on tumor sections (three samples per group) using a primary antibody against Ki-67 (1:500, Servicebio, Wuhan, China), and stained sections were imaged under a digital pathology slide scanner (Servicebio, Wuhan, China). For quantification, six fields were randomly selected per sample for statistical analysis.

### 2.12. Dual-Luciferase Reporter Assays

Candidate miRNA mimics and the corresponding negative control (mimics-NC), as well as the miRNA inhibitor and corresponding negative inhibitor control (inhibitor-NC), were synthesized by GenePharma (Suzhou, China). For target miRNA verification, miR-135a-5p mimics or miR-138-5p mimics and wild-type psiCHECK-2-AC092718.4 were co-transfected into 22RV1 cells. To identify the binding of each lncRNA and target miRNA, wild-type psiCHECK-2-AC092718.4 or mutant psiCHECK-2-AC092718.4 were co-transfected into 22RV1 cells. Then, 48 h post-transfection, cells were harvested. The Firefly and *Renilla* luciferase activities were analyzed using the Dual Luciferase Reporter Gene Assay Kit (Yeasen, Shanghai, China) on a GloMax^TM^ 96 microplate luminometer (Promega, Madison, WI, USA).

### 2.13. Statistical Analysis and Reproducibility

All experiments were performed in triplicate biological replicates at minimum. Student’s *t*-test and ANOVA (analysis of variance) were used for assessing statistical significance, as appropriate. *p* < 0.05 was considered statistically significant, and the results are presented with the following notations: ns, not significant; *, *p* < 0.05; **, *p* < 0.01; ***, *p* < 0.001.

## 3. Results

### 3.1. DHT-Induced DElncRNAs in LNCaP Cells

**Figure 1:**
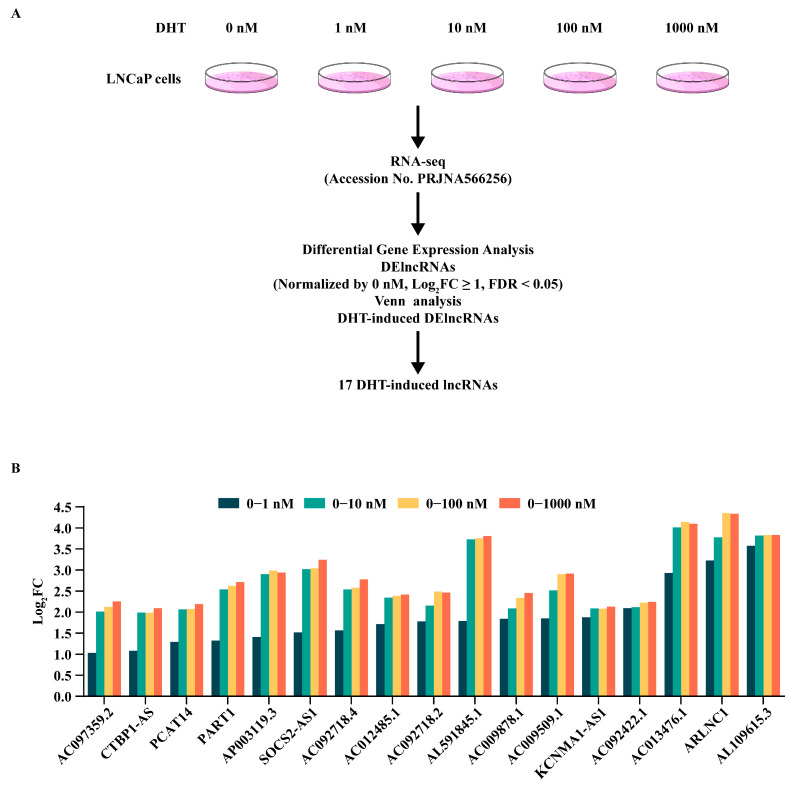
Screening of lncRNAs and expression profile induced by DHT in LNCaP cells. (**A**) Flow diagram for the identification of DHT-induced DElncRNAs in LNCaP cells. (**B**) Expression profile of DElncRNAs. The fold changes were normalized relative to the 0 nM group and are presented as log_2_FC. N = 3 biological replicates each group.

Androgens and lncRNAs function as fundamental elements in prostate development and the carcinogenesis of prostate cancer. As depicted in Figure 1A, to delve into the pathogenic molecular mechanisms of prostate cancer under the influence of androgens, in our previous study, we conducted transcriptome sequencing on the AR^+^ prostate cell line LNCaP, which was treated with 0 nM, 1 nM, 10 nM, 100 nM, and 1000 nM of DHT, respectively [8]. In our study, lncRNAs that overlapped across all concentrations and were upregulated by DHT were defined as DHT-induced DElncRNAs. Analysis of the RNA-seq data revealed that 17 DElncRNAs were significantly induced by DHT stimulation; the normalized fold changes in DHT-induced DElncRNAs are presented in Figure 1B and Supplementary Table S4. We found that some of these lncRNAs have been reported to be closely associated with prostate cancer, such as CTBP1-AS [16], PCAT14 [43], PART1 [44], SOCS2-AS1 [45], and ARLNC1 [46]. More importantly, some DHT-induced DElncRNAs identified in this study have been proven to be highly regulated in prostate cancer in an androgen-dependent manner, including CTBP1-AS [16], PART1, and SOCS2-AS1 [13].

Notably, AC092718.4 was also significantly upregulated in a DHT-induced manner, a novel finding that, to our knowledge, has not been previously reported. AC092718.4 is situated within intron 4 of the *Homo sapiens* centromere protein N (*CENPN*) gene, spanning the genomic region Chr16: 81, 030, 770–81, 031, 485 (Supplementary Figure S1A). Similar to most other lncRNAs, AC092718.4 was predicted, using an online tool, to lack protein-coding potential (Supplementary Figure S1B). The FISH assay demonstrated that this lncRNA is localized in the cytoplasm (Supplementary Figure S1C). Regarding biological functions, the LncACTdb 3.0 database predicted that AC092718.4 exhibits several cancer hallmarks, including self-sufficiency in growth signals, reprogramming of energy metabolism, and sustained angiogenesis (Supplementary Figure S1D). More importantly, bioinformatic analysis predicted that AC092718.4 is linked to a broad spectrum of malignancies, particularly prostate cancer (Supplementary Figure S1E).

### 3.2. The Expression File of DHT-Induced lncRNA AC092718.4 in Prostate Cancer

A previous study has revealed that AC092718.4 is significantly upregulated in various types of cancer and is especially highly expressed in lung adenocarcinoma [47]. It is associated with poor overall survival and disease-specific survival, having the potential to serve as a prognostic biomarker and promoting the progression of lung adenocarcinoma [47]. However, its potential role in the progression of prostate cancer, particularly in relation to the androgen response, remains elusive.

**Figure 2:**
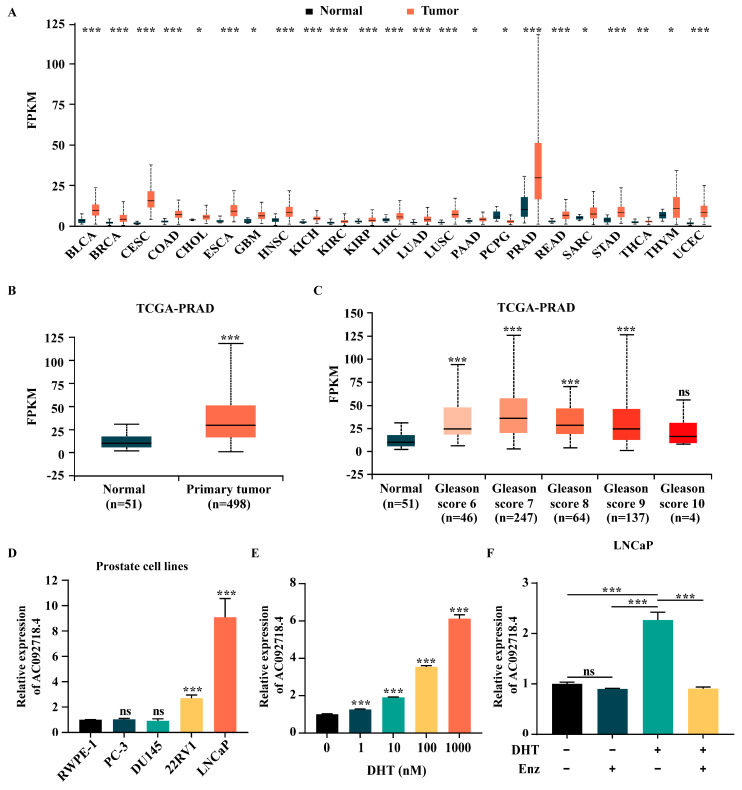
The expression of AC092718.4 in prostate cancer. (**A**) The expression profile of AC092718.4 across various cancer types, obtained from UALCAN database. BLCA, bladder urothelial carcinoma; BRCA, breast invasive carcinoma; CESC, cervical squamous cell carcinoma; COAD, colon adenocarcinoma; CHOL, cholangiocarcinoma; ESCA, esophageal carcinoma; GBM, glioblastoma multiforme; HNSC, head and neck squamous cell carcinoma; KICH, kidney chromophobe; KIRC, kidney renal clear cell carcinoma; KIRP, kidney renal papillary cell carcinoma; LIHC, liver hepatocellular carcinoma; LUAD, lung adenocarcinoma; LUSC, lung squamous cell carcinoma; PAAD, pancreatic adenocarcinoma; PCPG, pheochromocytoma and paraganglioma; PRAD, prostate adenocarcinoma; READ, rectum adenocarcinoma; SARC, sarcoma; STAD, stomach adenocarcinoma; THCA, thyroid carcinoma; THYM, thymoma; UCEC, uterine corpus endometrial carcinoma. (**B**) Comparative analysis of AC092718.4 expression between normal and tumor tissues in prostate cancer, obtained from UALCAN database. (**C**) Association between AC092718.4 expression and Gleason score, obtained from UALCAN database. (**D**) Endogenous expression of AC092718.4 in multiple prostate cell lines using RT-qPCR. (**E**) Validation of AC092718.4 expression under different DHT concentrations using RT-qPCR in LNCaP cells. (**F**) Effect of AR on AC092718.4 expression after treatment with DHT (10 nM) and/or enzalutamide (10 mM) in LNCaP cells was detected by RT-qPCR. Enz, enzalutamide. Data obtained from UALCAN database using Welch’s *t*-test. Data are presented as mean ± SD; n = 3 biological replicates. ns, not significant; *, *p* < 0.05; **, *p* < 0.01; ***, *p* < 0.001.

As depicted in Figure 2A, analysis of TCGA data revealed that AC092718.4 was most highly expressed in prostate tissue compared to all other normal tissues examined. Broad and upregulated expression of AC092718.4 was observed across multiple cancer types; for example, breast invasive carcinoma (BRCA), cervical squamous cell carcinoma (CESC), and prostate adenocarcinoma (PRAD). Notably, its expression was significantly higher in prostate cancer tissues compared to normal tissues (*p*-value = 2.117 × 10^−12^) (Figure 2B). Histological Gleason grading of prostate cancer demonstrated that AC092718.4 expression was significantly increased in the Gleason score 6–9 group relative to the normal control group (Figure 2C). RT-qPCR analysis further demonstrated that AC092718.4 was expressed at higher levels in AR^+^ cell lines (22RV1 and LNCaP) than in the normal prostate cell line (RWPE-1) and AR-negative (AR^-^) cell lines (PC-3 and DU145) (Figure 2D), suggesting that androgen responsiveness is consistent with the sequencing analysis results. Additionally, RT-qPCR results confirmed that AC092718.4 exhibited significant, dose-dependent upregulation following DHT stimulation in LNCaP cells (Figure 2E). Further, in order to investigate whether AC092718.4 is directly or indirectly regulated by AR, LNCaP cells were treated with DHT and/or enzalutamide, a second-generation androgen antagonist [48]. As shown in Figure 2F, AC092718.4 was upregulated in response to DHT and presented a slight but not significant decrease under enzalutamide. Interestingly, this promotion by DHT was counteracted by enzalutamide. Collectively, these results indicated that the DHT-induced lncRNA AC092718.4 was highly expressed in prostate cancer and may contribute to the pathogenesis and progression of prostate cancer.

### 3.3. The Effect of AC092718.4 on Cell Proliferation and Cell Apoptosis In Vitro

**Figure 3:**
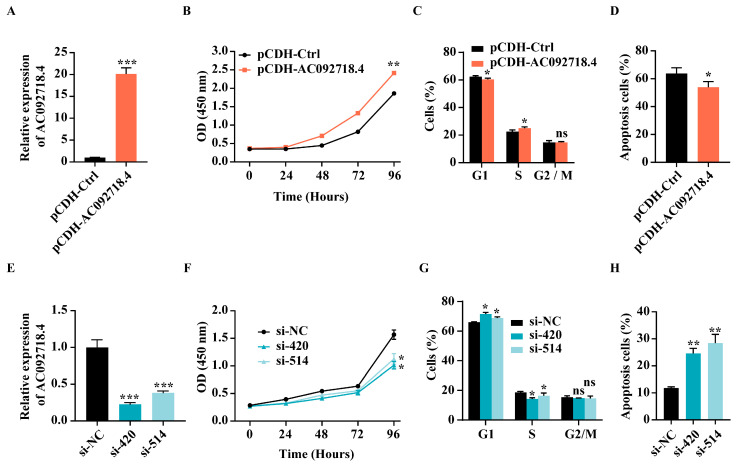
The effect of AC092718.4 on cell proliferation and apoptosis in prostate cancer cells in vitro. (**A**) Detection of AC092718.4 overexpression in LNCaP cells via RT-qPCR. (**B**) Assessment of cell proliferation with AC092718.4 overexpression in LNCaP cells at different time points using the CCK-8 assay. (**C**) Analysis of cell cycle transition in LNCaP cells with AC092718.4 overexpression by PI staining flow cytometry. (**D**) Analysis of cell cycle apoptosis in 22RV1 cells with AC092718.4 overexpression via Annexin V-YSFluor^TM^ 647/PI staining flow cytometry. (**E**) Detection of AC092718.4 knockdown in LNCaP cells via RT-qPCR. (**F**) Evaluation of cell proliferation in LNCaP cells with AC092718.4 knockdown using the CCK-8 assay. (**G**) Examination of cell cycle transition in LNCaP cells with AC092718.4 knockdown via PI staining flow cytometry. (**H**) Measurement of cell apoptosis in LNCaP cells with AC092718.4 knockdown using PI-FITC staining flow cytometry. Data are presented as mean ± SD; n = 3 biological replicates. ns, not significant; *, *p* < 0.05; **, *p* < 0.01; ***, *p* < 0.001.

To explore the role of DHT-induced lncRNA AC092718.4 in prostate cancer, the AR^+^ prostate cancer cell lines LNCaP and 22RV1 were chosen for a series of experimental assays. The overexpression efficiency of AC092718.4 was assessed via RT-qPCR. It was found that the expression level of AC092718.4 in the overexpressing group (pCDH-AC092718.4) was over 20-fold higher than that in the control group (pCDH-Ctrl) (Figure 3A). As shown in Figure 3B, the CCK-8 assay results revealed that overexpression of AC092718.4 significantly promoted cell proliferation, as evidenced by consistently higher absorbance values in the overexpressing group versus controls across multiple time points, suggesting sustained enhancement of cell growth. Subsequently, a PI staining flow cytometry assay revealed that overexpression of AC092718.4 significantly altered cell cycle distribution patterns (Figure 3C). Compared to the control group, the overexpressing group exhibited a notable reduction in the proportion of G1 phase cells, accompanied by a concurrent increase in S phase cells, while the G2/M phase population remained relatively unchanged, demonstrating that overexpression of AC092718.4 significantly accelerated the G1/S cell cycle transition (Figure 3C). Moreover, an Annexin V-YSFluor^TM^ 647/PI staining flow cytometry assay suggested that overexpression of AC092718.4 suppressed cell apoptosis (Figure 3D). Conversely, when AC092718.4 was knocked down by transfecting two specific siRNAs (si-420 and si-514) (Figure 3E), subsequent functional assays revealed that this knockdown led to a substantial decrease in cell proliferation capacity compared to the negative control (si-NC) (Figure 3F,G). As depicted in Figure 3F, the AC092718.4-knockdown groups exhibited a marked and time-dependent reduction in absorbance values compared to the negative control via CCK-8 assay, indicating significantly impaired cell proliferation capacity. PI staining flow cytometry analysis of cell cycle distribution further revealed that AC092718.4 depletion caused a pronounced accumulation of cells in the G1 phase, accompanied by a corresponding decline in the proportion of cells progressing to the S phase (Figure 3G), indicating an inhibition of the G1/S transition. Additionally, as depicted in Figure 3H, AC092718.4 knockdown significantly enhanced the rate of cell apoptosis, as evidenced by an increased percentage of apoptotic cells. These findings collectively demonstrate that AC092718.4 plays a pivotal role in promoting cell proliferation, facilitating G1/S cell cycle transition and suppressing cell apoptosis in prostate cancer in vitro.

### 3.4. The Effect of AC092718.4 on Tumorigenesis In Vivo

**Figure 4:**
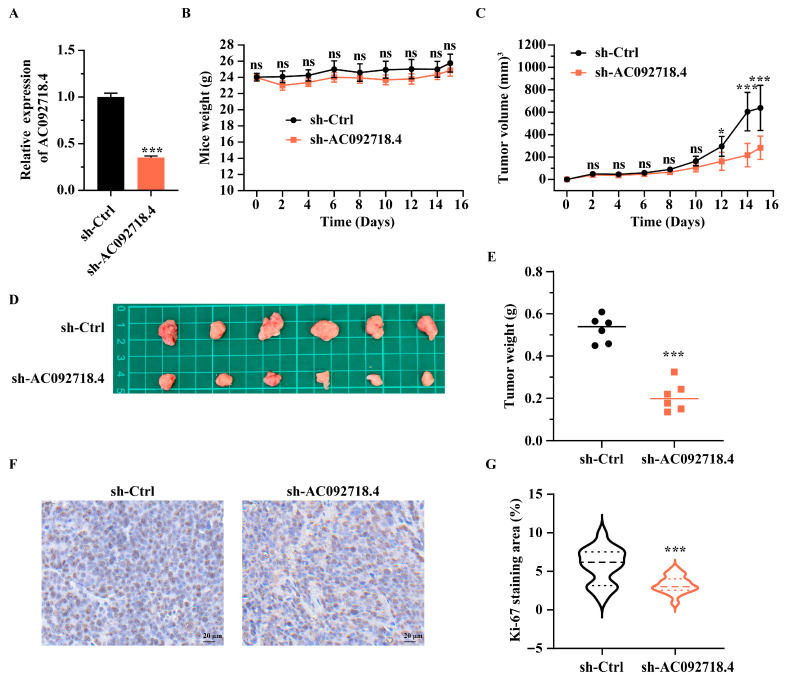
Knockdown of AC092718.4 suppresses tumorigenesis in vivo. (**A**) Detection of stable knockdown efficiency of AC092718.4 in 22RV1 cells via RT-qPCR. Measurement of body weight (**B**) and tumor volume (**C**) from xenograft models in nude mice. (**D**) Representative tumor image from nude mice implanted with cancer cells transfected with sh-Ctrl or sh-AC092718.4. (**E**) Measurement of tumor weight from xenograft models in nude mice. (**F**) Representative images of Ki-67 staining in tumor tissues obtained from nude mice. (**G**) Analysis of Ki-67 expression via immunohistochemical staining. Data are presented as mean ± SD; n = 6 biological replicates for tumor monitor; n = 3 biological replicates for Ki-67 staining. ns, not significant; *, *p* < 0.05; ***, *p* < 0.001.

To examine the involvement of AC092718.4 in tumorigenesis in vivo, a xenograft tumor assay was performed. Given that the tumorigenicity of the AR^+^ 22RV1 cell line is superior to that of the LNCaP cell line [49,50], the 22RV1 cell line was selected. The endogenous expression of AC092718.4 in 22RV1 cells was stably downregulated by lentiviral shRNAs (sh-AC092718.4) to approximately 60% of that in the control group (sh-Ctrl) (Figure 4A). The xenograft tumor assay revealed that, without influencing the body weight of mice (Figure 4B), knockdown of AC092718.4 significantly suppressed tumor growth in mice, as evidenced by tumor volume (Figure 4C) and tumor weight (Figure 4D,E). Meanwhile, the immunohistochemistry assay demonstrated that the expression levels of the proliferation-associated protein Ki-67 in tumor tissues were significantly lower than those in the negative control (Figure 4F,G).

Altogether, our data indicate that the lncRNA AC092718.4, induced by DHT, exhibits high expression levels in prostate cancer tissues and AR^+^ prostate cancer cell lines. Notably, the elevated expression of AC092718.4 promotes cell proliferation and cell cycle progression. Conversely, its downregulation facilitates cell apoptosis in vitro and hinders tumorigenesis in vivo.

### 3.5. The Interaction Between AC092718.4 and miR-138-5p

**Figure 5:**
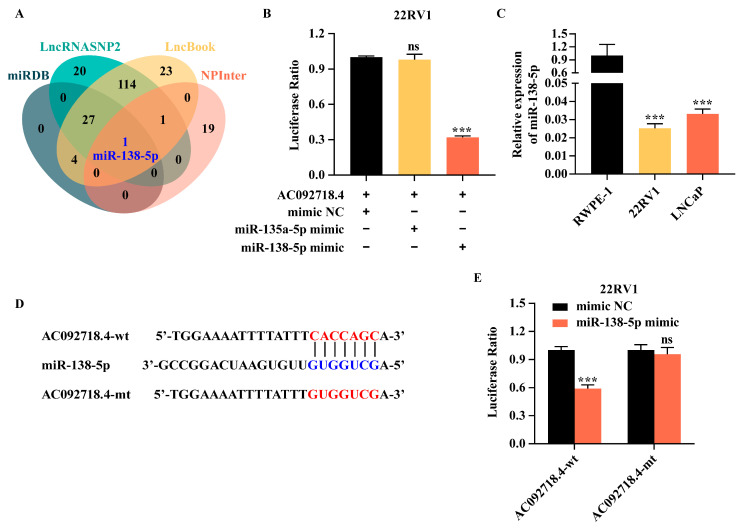
AC092718.4 acts as a sponge for miR-138-5p. (**A**) Putative binding miRNAs of AC092718.4 were analyzed using four online databases, and the overlapping miRNA was highlighted in blue. (**B**) A dual-luciferase assay was conducted to identify the downstream miRNAs of AC092718.4. Plasmids containing AC092718.4 and miR-135a-5p or miR-138-5p mimics were transfected into 22RV1 cells. (**C**) Endogenous miR-138-5p was detected via RT-qPCR in different prostate cell lines. (**D**) A schematic diagram illustrates the putative binding sites between AC092718.4 and miR-138-5p. The strategy for constructing the AC092718.4 mutant is also presented. wt: wild-type; mt: mutant. (**E**) A dual-luciferase reporter assay was carried out to detect the binding between miR-138-5p and AC092718.4. Plasmids containing wild-type or mutant AC092718.4 and miR-138-5p mimics were transfected into 22RV1 cells. Data are presented as mean ± SD; n = 3 biological replicates. ns, not significant; ***, *p* < 0.001.

LncRNAs can function as ceRNAs by sponging miRNAs, thereby regulating downstream gene expression [19,51]. To identify miRNAs that potentially interact with AC092718.4, we conducted a bioinformatic screening using four prediction databases. Venn analysis revealed that miR-138-5p was the only miRNA overlapping across all databases, suggesting that it may be a candidate miRNA sponged by AC092718.4 (Figure 5A). Given that AC092718.4 has been reported to bind to miR-135a-5p in breast cancer [52], we further investigated whether miR-138-5p and/or miR-135a-5p interacts with AC092718.4 in prostate cancer. The dual-luciferase reporter assay confirmed that a specific interaction occurs between AC092718.4 and miR-138-5p but not with miR-135a-5p in 22RV1 cells (Figure 5B).

Interestingly, the expression levels of miR-138-5p in AR^+^ cell lines were lower than in the normal cell line (Figure 5C), showing an inverse correlation with the levels of AC092718.4. To determine whether the lncRNA AC092718.4 functions as a ceRNA for miR-138-5p, we identified a putative binding site for miR-138-5p at the 3’UTR of AC092718.4 (AC092718.4-wt) and generated a corresponding binding-site mutant (AC092718.4-mt) (Figure 5D). As shown in Figure 5E, luciferase activity was significantly reduced when miR-138-5p mimics were co-transfected with plasmid containing the wild-type AC092718.4 (AC092718.4-wt), whereas no notable change was observed with plasmid containing the mutant (AC092718.4-mt) (Figure 5E). The above results demonstrate that AC092718.4 directly binds to miR-138-5p and acts as its molecular sponge in AR^+^ prostate cancer cells.

### 3.6. The ceRNA Axis Regulated AC092718.4 in Prostate Cancer Cells

**Figure 6:**
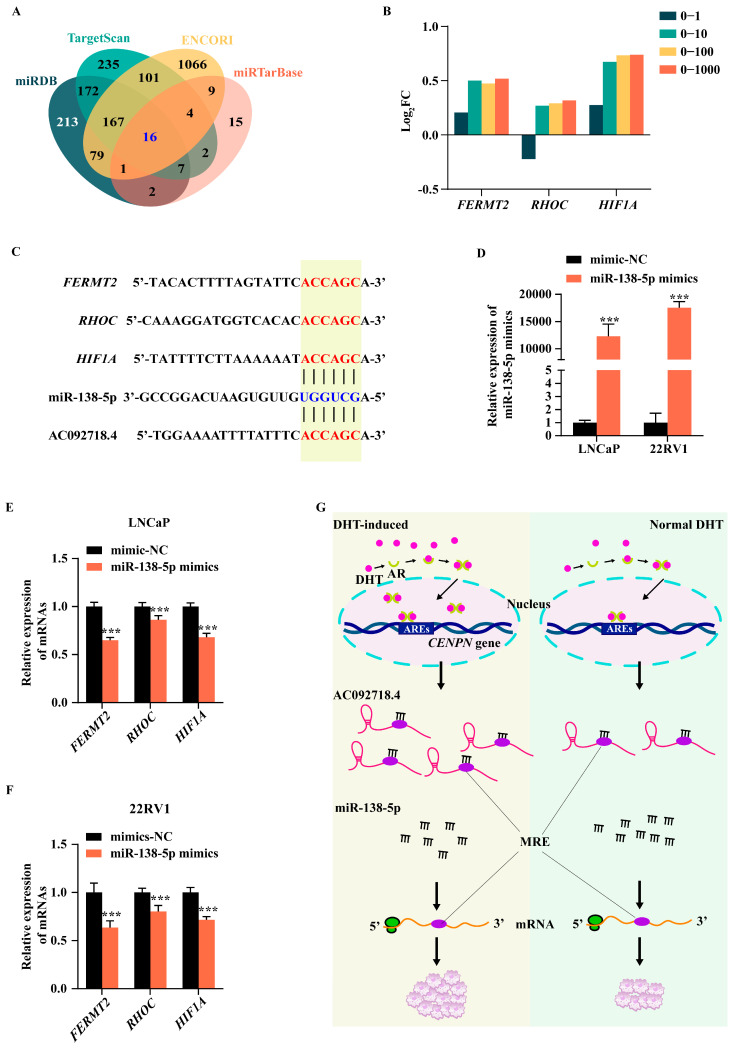
MiR-138-5p downregulates DHT-induced mRNAs. (**A**) Putative target mRNAs of miR-138-5p were analyzed using four online databases. The overlapping mRNAs are highlighted in blue. (**B**) The DHT-induced mRNAs were analyzed based on RNA-seq data. (**C**) A schematic diagram illustrates the putative binding sites between candidate genes and miR-138-5p, the strategy for constructing mRNA mutations is also presented. (**D**) Expression of miR-138-5p was detected via RT-qPCR. The expression levels of downstream genes under the condition of miR-138-5p overexpression were measured by RT-qPCR in LNCaP (**E**) and 22RV1 (**F**) cells. (**G**) A proposed model for the AC092718.4-mediated regulation of AR^+^ prostate cancer cell proliferation via ceRNA mechanism. Data are presented as mean ± SD; n = 3 biological replicates. ***, *p* < 0.001.

MiRNAs post-transcriptionally regulate cancer-associated genes by triggering downstream mRNA degradation or inhibiting translation [53]. To identify downstream targets of miR-138-5p, we first performed bioinformatic screening using four prediction databases. Venn analysis revealed 16 candidate genes are common to all databases (Figure 6A and Supplementary Table S5), suggesting that they may be directly inhibited by miR-138-5p. To focus on androgen-responsive pathways in prostate cancer, we integrated these candidates with DEmRNAs upregulated by DHT treatment. This intersection yielded three DHT-induced mRNAs (*FERMT2*, *RHOC*, and *HIF1A*) (Figure 6B and Supplementary Table S6). Importantly, these mRNAs are well-established contributors to prostate cancer progression. *FERMT2* is highly expressed in prostate tumors and promotes cell adhesion and migration [22]. *RHOC* is mainly involved in prostate cancer migration and metastasis; notably, RhoC-targeted vaccination has been proposed as a promising therapy for delaying or preventing tumor recurrence and metastasis formation [54,55]. *HIF1A* is closely related to both hypoxia response and AR signaling in prostate cancer. Under hypoxic conditions, the HIF1a protein becomes stabilized, which in turn upregulates AR expression. Independently, HIF1a can also promote prostate cancer progression even in the absence of AR signaling [56].

The inhibitory effect of miRNA on downstream genes is accomplished by binding to the 3′UTR of mRNAs and promoting their degradation through the recruitment of the RNA-induced silencing complex [19]. Therefore, we analyzed the binding between miR-138-5p and the three candidate genes. As expected, miR-138-5p could theoretically bind to the untranslated region of the three candidate target genes (Figure 6C). Indeed, *FERMT2* has been confirmed to participate in prostate cancer progression via miR-138-5p-mediated inhibition [57]. Moreover, our results revealed that overexpression of miR-138-5p via miR-138-5p mimics transfection (Figure 6D), significantly downregulating all three candidate genes in LNCaP and 22RV1 cells (Figure 6E,F). In contrast, transfection of the miR-138-5p inhibitor had almost no impact on these candidates (Supplementary Figure S2), likely because the low endogenous expression level of miR-138-5p exerted negligible influence on downstream genes. Meanwhile, considering that AC092718.4 can also be perfectly matched with the seed sequence of miR-138-5p through the same core sequence as mRNAs (Figure 6C), our findings support a working model in which a DHT-induced lncRNA promotes the proliferation of prostate cancer cells via the AC092718.4/miR-138-5p/mRNA axes. As summarized in Figure 6G, DHT-activated AR signaling upregulates lncRNA AC092718.4, which in turn functions as a ceRNA and sequesters miR-138-5p. This sequestration relieves miR-138-5p-mediated repression of oncogenic mRNAs including *FERMT2*, *RHOC*, and *HIF1A*. In terms of mechanism, this could potentially form three corresponding ceRNA axes, the activation of which would lead to an increase in downstream mRNA expression and ultimately drive the proliferation of prostate cancer cells.

## 4. Discussion

The pathogenesis and progression of prostate cancer are heavily dependent on aberrant AR signaling. Fluctuations in androgen levels can induce substantial alterations in the transcriptome. Given that patients often develop resistance to conventional AR-targeted therapies, genetic interventions are emerging as a promising therapeutic approach [9]. The ceRNA network, as a typical post-regulatory pattern, has been extensively studied in various types of cancer [9,17,19]. It is anticipated to serve as a novel gene intervention strategy, particularly in precision medicine.

By treating the AR^+^ cell line LNCaP with different concentrations of DHT and performing transcriptome sequencing analysis, we identified numerous DHT-induced lncRNAs. Notably, we identified a novel DHT-induced lncRNA AC092718.4, which exhibited a marked dependency on the DHT dose. Through analyzing the TCGA data, we also found that AC092718.4 exhibited the highest expression in normal prostate across all the normal organs or tissues (Figure 2A), suggesting that AC092718.4 not only responds to androgen signals but may also play an important role in normal prostate function. AC092718.4 has been documented in multiple cancers. AC092718.4 is highly expressed in lung cancer cell lines and may serve as a reliable prognostic biomarker in lung adenocarcinoma [47,58]. A model proposing AC092718.4 as a ceRNA has also been suggested. It may function as a ceRNA to competitively bind to miR-135a-5p, thereby upregulating S100P expression and promoting breast cancer cells’ resistance to trastuzumab [52]. Additionally, AC092718.4 is involved in the ceRNA network associated with CD8^+^ T cell infiltration in breast cancer [59]. Furthermore, AC092718.4 participates in the response of childhood cancers to high-dose ionizing radiation by regulating cell cycle regulation and DNA damage response, as well as interacting with miRNAs [60]. In the present study, we verified that AC092718.4 not only displayed DHT dose-dependent expression but also showed higher expression in AR^+^ cell lines compared to normal cell line and AR^-^ cell lines, highlighting the impact of androgen and AR on AC092718.4.

Additionally, TCGA data revealed that AC092718.4 is upregulated in prostate cancer compared to the normal tissues (Figure 2A,B). A series function prediction revealed that it possesses remarkable cancer hallmarks (Supplementary Figure S1D) and is associated with prostate cancer (Supplementary Figure S1E), indicating that AC092718.4 may be involved in prostate cancer. Simultaneously, the Gleason grading indicates that AC092718.4 expression is higher in patients with Gleason scores of 6–9 than in a normal control group (Figure 2C). Previous studies have demonstrated that AC092718.4 promotes cell invasion, migration, and proliferation in lung adenocarcinoma [47,58]. Our study found that overexpression of AC092718.4 promoted cell proliferation, accelerated cell cycle transition, and suppressed cell apoptosis in AR^+^ prostate cancer cells (Figure 3A–D). Conversely, knockdown of AC092718.4 inhibited cell proliferation and facilitated cell apoptosis in vitro (Figure 3E–H) and suppressed tumorigenesis in vivo (Figure 4). Our results indicated that AC092718.4 acts as an oncogene in prostate cancer, providing a novel target gene for genetic intervention therapy in prostate cancer.

LncRNAs and miRNAs serve as pivotal regulators of gene expression, being extensively and profoundly implicated in the critical steps of cancer pathogenesis [19]. Within the ceRNA network, lncRNAs act as sponges for miRNAs via MRE. AC092718.4 has been either predicted or experimentally validated to bind to miRNAs, such as miR-135a-5p in breast cancer [52]. In the present study, based on bioinformatic analysis, miR-138-5p was chosen as a candidate target miRNA (Figure 5A). Further analysis supported that AC092718.4 bound to miR-138-5p but not to miR-135a-5p in prostate cancer cells (Figure 5B).

Mature human miR-138-5p (also referred to as hsa-miR-138) originates from two precursors, hsa-mir-138-1 and hsa-mir-138-2, as indicated in the miRDB database. These precursor miRNAs are genomically located on chromosomes 3p21.32 and 16q13, respectively, and collectively constitute the miR-138 family [61]. MiR-138-5p is regarded as tumor suppressor, and downregulation of miR-138-5p is frequently observed in multiple cancer types, including prostate cancer, colorectal cancer, head and neck squamous cell carcinoma, anaplastic thyroid carcinoma, non-small-cell lung cancer, oral squamous cell carcinoma, and tongue squamous cell carcinoma [62,63,64]. In the context of cell lines, previous studies have revealed that expression of miR-138-5p is lower in human prostate cancer cell lines compared to the prostate epithelial cell line RWPE-1 [62,65]. Interestingly, its expression in CRPC cell lines and AR^-^ cell lines (PC-3 and DU145) is even lower than in AR^+^ prostate cancer cell lines (LNCaP and 22RV1) [62]. Importantly, its content is simultaneously negatively correlated with the Gleason score, lymph node metastasis, and poor prognosis in prostate cancer. Overexpression of miR-138-5p inhibits the malignant progression of prostate cancer [62]. In the present study, the endogenous expression of miR-138-5p was shown to be lower in AR^+^ cell lines than in the normal cell line (Figure 5C), which is consistent with previous research findings. The dual-luciferase reporter assay further confirmed that AC092718.4 can bind to wild-type miR-138-5p but not the mutant (Figure 5D,E), suggesting that AC082718.4 could act as an miRNA sponge for miR-138-5p.

Determining the lncRNA/miRNA axis is crucial for assessing targetability [19]. MiRNAs can exert an oncogenic role by inhibiting tumor-suppressor genes, while they demonstrate anti-oncogenic potential by suppressing proto-oncogenes [53]. It is widely recognized that miR-138-5p is downregulated in tumors or cancer cells, functioning as a tumor suppressor by targeting oncogenes to impede cancer progression [63,66,67,68]. In this study, through bioinformatic analysis and comprehensive consideration of the effect of DHT, three downstream candidate mRNAs (*FERMT2*, *RHOC*, and *HIF1A*) were selected (Figure 6A,B). Moreover, the expression levels of these genes were significantly downregulated upon transfection with miR-138-5p mimics, with *FERMT2* showing the most pronounced downregulation. However, the miR-138-5p inhibitor did not distinctly affect gene expression, which might be due to the low endogenous expression of miR-138-5p in AR^+^ cell lines (Figure 6D–F).

The *FERMT2* gene encodes *Kindlin-2* (also known as *K2* or *Mig-2*), an integrin-binding protein essential for integrin activation [22,69]. *FERMT2* is highly expressed in many cancers, particularly in prostate cancer [22]. It regulates the adhesion, spreading, migration, and tumorigenesis of prostate cancer cells [22]. Moreover, *FERMT2* is involved in the sensitivity of metastatic castration-resistant prostate cancer to chemotherapeutics via the miR-138/K2/β1-integrin signaling axis [57]. These key roles indicate that *FERMT2* may be an interesting therapeutic target for treating prostate cancer [22].

The *RHOC* gene encodes RhoC protein, a member of the Rho GTPase family, which mediates the cell migration and invasion processes of various tumors, such as prostate cancer, melanoma, and inflammatory breast cancer, and may potentially serve as a new target for anti-metastasis therapy [54,70,71]. Although there are currently no drugs or therapies directly targeting the *RHOC* gene, several studies have revealed that a reduction in its activity or inhibition of *RHOC* expression through siRNA, antibodies or small-molecule inhibitors could impede cancer progression, demonstrating the enormous potential of RHOC as a therapeutic target [72].

The *HIF1A* gene and its encoded HIF1a protein play pivotal roles in cancer initiation and progression. Single nucleotide polymorphisms in the *HIF1A* gene have been associated with 14 types of cancer, notably prostate, breast, and lung cancers [73]. The HIF1a subunit dimerizes with the HIF1b subunit to form an active HIF transcription complex, which then translocates to the nucleus, leading to the transcription of cancer-related genes [56]. In prostate cancer, HIF1A not only binds to AR to promote tumor growth but also has the ability to restore tumor growth in the absence of AR signaling [56].

In the present study, we propose that there are AC092718.4/miR-138-5p/mRNA axes in prostate cancer, and that AC092718.4 and the three target genes competitively bind to miR-138-5p (Figure 6G). Considering that DHT induced expression of AC092718.4, when studying the regulatory effects among the three molecules, DHT may be a potential regulatory participant, and the inhibitory effect of miR-138-5p on target genes may be alleviated when AC092718.4 is upregulated by DHT stimulation.

Although several studies have demonstrated that AC092718.4 is a widely expressed gene across multiple types of cancer and suggested that it could serve as a prognostic biomarker, this gene remains poorly understood. In the present study, combining bioinformatic analysis with biological experiments, we found that AC092718.4, which is most highly expressed in prostate tissue, is upregulated by DHT in AR^+^ prostate cancer cell line. Functionally, ectopic overexpression of AC092718.4 significantly promotes the growth of prostate cancer cells, while its downregulation facilitates cell apoptosis and attenuates tumorigenesis. Mechanistically, both AC092718.4 and the three target genes (*FERMT2, RHOC, HIF1A*) competitively bind to miR-138-5p (Figure 6C), forming the AC092718.4/miR-138-5p/mRNA axes (Figure 6G). The inhibitory effect of miR-138-5p on target genes may be alleviated when AC092718.4 is upregulated by DHT stimulation, thereby leading to the upregulation of the three oncogenes, ultimately promoting cell proliferation. Overall, the newly proposed AC092718.4/miR-138-5p/mRNA axes are closely associated with androgens, which might represent a promising target for overcoming androgen therapy resistance in prostate cancer.

## 5. Conclusions

Understanding the physiological fluctuations that occur in response to alterations in androgen levels holds significant importance in prostate cancer therapy, particularly following the failure of ADT. Consequently, exploring the androgen-induced signaling axis or network represents a promising research orientation. In our study, we discovered that a novel prostate-cancer-related lncRNA, AC092718.4, is closely associated with prostate cancer and functions as an miRNA sponge to bind to miR-138-5p, thereby regulating the downstream oncogenic mRNAs. This lncRNA, along with its target miRNA miR-138-5p and downstream mRNAs, has the potential to establish a ceRNA axis. This axis plays a role in prostate cancer and has the potential to become a target for gene therapy.

## Data Availability

The data supporting the current findings are contained within the manuscript. Additional original data are available from the corresponding author upon reasonable request.

## Supplementary Materials

The following supporting information can be downloaded at: https://www.mdpi.com/article/10.3390/genes17050538/s1, Supplementary Figure S1: The molecular characteristics of AC092718.4; Supplementary Figure S2: The effect of miR-138-5p inhibitor on the expression of target mRNAs; Supplementary Table S1: The primer pairs used in this study; Supplementary Table S2: The probes used in FISH assay; Supplementary Table S3: The sequences of siRNAs and shRNAs used in this study; Supplementary Table S4: The normalized log_2_FC of DHT-induced DElncRNAs; Supplementary Table S5: The candidate target genes of miR-138-5p predicted by bioinformatic analysis; Supplementary Table S6: The normalized log_2_FC of miR-138-5p target mRNAs (the DHT-induced mRNAs are marked in red).

## Abbreviations

The following abbreviations are used in this manuscript:

DHT	dihydrotestosterone
ADT	androgen deprivation therapy
CRPC	castration-resistant prostate cancer
PCA3	prostate cancer antigen 3
FDA	Food and Drug Administration
ceRNAs	competing endogenous RNAs
MREs	miRNA recognition elements
DElncRNAs	differentially expressed lncRNAs
CS-FBS	charcoal-stripped fetal bovine serum
Enz	enzalutamide
SRA	Sequence Read Archive
DEGs	differentially expressed genes
FDR	false discovery rate
DEmRNAs	differentially expressed mRNAs
RT-qPCR	Reverse-Transcription Quantitative PCR
FISH	fluorescence in situ hybridization
DAPI	4′,6-diamidino-2-phenylindole
CCK-8	Cell Counting Kit-8
ANOVA	analysis of variance
IHC	immunohistochemistry
CENPN	Homo sapiens centromere protein N
BRCA	breast invasive carcinoma
CESC	cervical squamous cell carcinoma
PRAD	prostate adenocarcinoma
BLCA	bladder urothelial carcinoma
COAD	colon adenocarcinoma
CHOL	cholangiocarcinoma
ESCA	esophageal carcinoma
GBM	glioblastoma multiforme
HNSC	head and neck squamous cell carcinoma
KICH	kidney chromophobe
KIRC	kidney renal clear cell carcinoma
KIRP	kidney renal papillary cell carcinoma
LIHC	liver hepatocellular carcinoma
LUAD	lung adenocarcinoma
LUSC	lung squamous cell carcinoma
PAAD	pancreatic adenocarcinoma
PCPG	pheochromocytoma and paraganglioma
READ	rectum adenocarcinoma
SARC	sarcoma
STAD	stomach adenocarcinoma
THCA	thyroid carcinoma
THYM	thymoma
UCEC	uterine corpus endometrial carcinoma
