# Supplementary material for: DHT-Induced lncRNA AC092718.4 Promotes Prostate Cancer Cell Proliferation via ceRNA Mechanism
Source: Genes (Basel). 2026 May 1;17(5):538. doi: 10.3390/genes17050538 (PMC13205834; doi:10.3390/genes17050538)
Supplement: Supplementary file 1 [file genes-17-00538-s001.zip › Revised Supplymentary Materials (Clean version).pdf]

Supporting Information for

DHT-induced lncRNA AC092718.4 promotes prostate cancer cell proliferation via ceRNA mechanism

Lian Jin [1,2], Shan Feng [1,2], Wei-Jie Sun [2], Jun Ouyang [1,2], Feng Liu [1,2], Bai-Cheng Lu [1,2], Ya-Ping Zhang [2,3,4,*], Hui Zhao [1,2,*]

Correspondence: zhangyp@mail.kiz.ac.cn (Y.-P.Z.); zhaohui@ynu.edu.cn (H.Z.)

SUPPLEMENTARY FIGURE LEGENDS

Supplementary Figure S1. The molecular characteristics of AC092718.4. (A) The genomic location of AC092718.4. (**B**) The protein-coding capacity predicted by LncRBase database. (**C**) The cellular localization validated via FISH assay. (**D**) The cancer hallmarks predicted by LncACTdb 3.0 database. (**E**) The biological functions predicted by LncACTdb 3.0 database, prostate cancer-related data is marked in blue box.

Supplementary Figure S2. The effect of miR-138-5p inhibitor on the expression of target mRNAs. (**A**) Expression of miR-138-5p was detected via RT-qPCR. The expression levels of downstream mRNAs in LNCaP (B) and 22RV1 (**C**) were examined by RT-qPCR. ns, not significant; *, $p < 0.05$; ***, $p < 0.001$.

Supplementary Table S1. The primer pairs used in this study.

Primers	Usages	Sequences (5'→3')
ACTB-F	Quantification of AC092718.4	TGCGTCTGGACCTGGCTGG
ACTB-R		TAGCTCTTCTCCAGGGAGGA
AC092718.4-F		GAGGCTGAGGCAGGAGAATC
AC092718.4-R		CAAGTTGCAGGTCATGTAGGG
miR-138-5p-RT	Reverse transcription of miR-138-5p	GTCGTATCCAGTGCAGGGTCCGAGGTATTCGCACTGGATACGACCGGCCT
U6-F	Quantification of miR-138-5p	CGCTTCGGCAGCACATATAC
U6-R		TTCACGAATTTGCGTGTCAT
miR-138-5p-F		GCGAGCTGGTGTTGTGAATC
miR-138-5p-R		AGTGCAGGGTCCGAGGTATT
psiCHECK-2-PmeI-F	Linearization of psiCHECK-2 vector	GTTTAAACCTAGAGCGGCCGC
psiCHECK-2-XhoI-R		CTCGAGCGATCGCCTAGAATT
HR-AC092718.4-XhoI-F	Adding homologous arms for AC092718.4	attctaggcgatcgctcgagGCATGGTGGCTCACGCCT
HR-AC092718.4-PmeI-R		cggccgctctaggtttaaacGCAGCAAGACAAGGTGTTTTTATT
AC092718.4-mt-F	MREs binding sites mutation	TTgtggtcgACTACAACTCCTCAACAGCACCAA
AC092718.4-mt-R		GTTGTAGTcgaccacAAATAAAATTTTCCAGGATGCAGTG
FERMT2-F	Quantification of FERMT2	GTATGGTATTCAGGCAGATGCTAAG
FERMT2-R		TCGGGGTGTCTGATATTAAAAGTCT
RHOC-F	Quantification of RHOC	AAGATGAAGCAGGAGCCCGTT
RHOC-R		GAGCACTCAAGGTAGCCAAAGG
HIF1A-F	Quantification of HIF1A	CTCCATTACCCACCGCTGAA
HIF1A-R		ATTAGGCTCAGGTGAACTTTGTCT

Supplementary Table S2. The probes used in FISH assay.

Probes	Sequences (5’ → 3’)
18S	CTTCCTTGGATGTGGTAGCCGTTTC
AC092718.4	TAGCTGGGCCTGGTGGTGTGCAC

Supplementary Table S3. The sequences of siRNAs and shRNAs used in this study.

Names	Usages	Oligo sequences (5'→3')
si-420-sense	AC092718.4	UACAGGAGCUUACCUUGAATT
si-420-antisense	transiently	UUCAAGGUAAGCUCCUGUATT
si-514-sense	knockdown	CAGCACCAACCAAUAAACUTT
si-514-antisense		AGUUUAUUGGUUGGUGCUGTT
sh-420-F	AC092718.4	GATCCTACAGGAGCTTACCTTGAACTTCCTGTCAGATTCAAGGTAAGCTCCTGTATTTTTG
sh-420-R	stably	AATTCAAAAATACAGGAGCTTACCTTGAATCTGACAGGAAGTTCAAGGTAAGCTCCTGTAG
sh-514-F	knockdown	GATCCCAGCACCAACCAATAAACTCTTCCTGTCAGAAGTTTATTGGTTGGTGCTGTTTTTG
sh-514-R		AATTCAAAAACAGCACCAACCAATAAACTTCTGACAGGAAGAGTTTATTGGTTGGTGCTGG

Supplementary Table S4. The normalized log_2FC of DHT-induced DElncRNAs.

LncRNAs	DHT (nM)			
	**0−1**	**0−10**	**0−100**	**0−1000**
AC097359.2	1.03345894	2.013142409	2.124774916	2.253887293
CTBP1-AS	1.08042495	1.988854171	1.984653047	2.094226632
PCAT14	1.2907379	2.063599963	2.072249685	2.193157953
PART1	1.32228892	2.54079297	2.627346766	2.711780203
AP003119.3	1.40898679	2.902430783	2.986772153	2.938889903
SOCS2-AS1	1.51978532	3.021064035	3.038662857	3.24472389
AC092718.4	1.56640739	2.535983617	2.578130877	2.775992828
AC012485.1	1.71547056	2.343191929	2.386265014	2.417111906
AC092718.2	1.78124904	2.155899364	2.48937399	2.460115408
AL591845.1	1.79023905	3.729831739	3.756560137	3.808942487
AC009878.1	1.84446495	2.090104021	2.337616172	2.454967311
AC009509.1	1.85050484	2.512800328	2.899711337	2.915340103
KCNMA1-AS1	1.87632559	2.085958417	2.081519158	2.132053509
AC092422.1	2.09487086	2.11832598	2.22659891	2.24540818
AC013476.1	2.93192125	4.010468196	4.143409667	4.10066209
ARLNC1	3.22625777	3.775823572	4.350817878	4.335546795
AL109615.3	3.57548479	3.820582652	3.825371939	3.833474364

Supplementary Table S5. The candidate target genes of miR-138-5p predicted by bioinformatic analysis.

Genes	Gene IDs	Description
RARA	ENSG00000131759	retinoic acid receptor alpha
FOXC1	ENSG00000054598	forkhead box C1
AGO1	ENSG00000092847	argonaute RISC catalytic component 1
EID1	ENSG00000255302	EP300 interacting inhibitor of differentiation 1
ZEB2	ENSG00000169554	zinc finger E-box binding homeobox 2
CCND3	ENSG00000112576	cyclin D3
DNAJB6	ENSG00000105993	DnaJ (Hsp40) homolog, subfamily B, member 6
FERMT2	ENSG00000073712	fermitin family member 2
RHOC	ENSG00000155366	ras homolog family member C
ROCK2	ENSG00000134318	Rho associated coiled-coil containing protein kinase 2
EIF4EBP1	ENSG00000187840	eukaryotic translation initiation factor 4E binding protein 1
HIF1A	ENSG00000100644	hypoxia inducible factor 1, alpha subunit (basic helix-loop-helix transcription factor)
CASP3	ENSG00000164305	caspase 3
PPM1L	ENSG00000163590	protein phosphatase, Mg2+/Mn2+ dependent 1L
ZMYND11	ENSG00000015171	zinc finger MYND-type containing 11
IGF2BP1	ENSG00000159217	insulin like growth factor 2 mRNA binding protein 1

Supplementary Table S6. The normalized log_2FC of miR-138-5p target mRNAs (the DHT-induced mRNAs are marked in red).

DHT concentrations (nM)	Genes	Log_2FC	FC	padj
0-1	*RARA*	-0.81913	0.566783	2.70×10^{-19}
	FOXC1	0.483491	1.398123	8.47×10^{-1}
	AGO1	-0.12101	0.919542	8.50×10^{-3}
	EID1	-0.1931	0.874723	7.97×10^{-3}
	ZEB2	2.875814	7.340171	NA
	CCND3	-0.16849	0.889773	3.77×10^{-2}
	DNAJB6	-0.12122	0.919412	9.42×10^{-2}
	FERMT2	0.207862	1.154976	9.64×10^{-3}
	RHOC	-0.22339	0.856549	8.79×10^{-4}
	ROCK2	-0.02084	0.985658	8.71×10^{-1}
	EIF4EBP1	-0.14588	0.903829	1.80×10^{-1}
	HIF1A	0.276142	1.210953	1.78×10^{-12}
	CASP3	-0.31792	0.802225	6.73×10^{-1}
	PPM1L	-0.24993	0.840936	2.14×10^{-1}
	ZMYND11	-0.22298	0.856791	2.85×10^{-6}
0-10	*RARA*	-0.80064	0.574095	9.29×10^{-15}
	FOXC1	2.165829	4.487243	2.21×10^{-2}
	AGO1	-0.11813	0.921379	5.96×10^{-2}
	EID1	-0.01711	0.988212	8.99×10^{-1}
	ZEB2	3.291259	9.78966	NA
	CCND3	-0.45443	0.729798	6.20×10^{-2}
	DNAJB6	-0.02148	0.985223	8.53×10^{-1}
	FERMT2	0.501619	1.415801	4.69×10^{-7}
	RHOC	0.269492	1.205384	6.06×10^{-4}
	ROCK2	-0.05967	0.959484	5.69×10^{-1}
	EIF4EBP1	-0.46076	0.726606	9.21×10^{-9}
	HIF1A	0.674284	1.595804	3.85×10^{-17}
	CASP3	-0.09365	0.937151	7.35×10^{-1}
	PPM1L	0.136188	1.098997	4.83×10^{-1}
	ZMYND11	-0.3598	0.779271	6.94×10^{-9}
	IGF2BP1	2.969114	7.83055	NA
0-100	*RARA*	-0.77583	0.584052	4.38×10^{-11}
	FOXC1	2.111223	4.320575	1.97×10^{-2}
	AGO1,AGO3	-0.05688	0.961341	4.63×10^{-1}
	EID1	-0.00959	0.993375	9.36×10^{-1}
	CCND3	-0.14303	0.905615	2.02×10^{-1}
	DNAJB6	-0.02332	0.983965	8.29×10^{-1}

	FERMT2	0.474473	1.38941	1.48×10^{-8}
	RHOC	0.29019	1.222801	2.58×10^{-3}
	ROCK2	-0.05198	0.964612	6.10×10^{-1}
	EIF4EBP1	-0.46232	0.725817	3.00×10^{-9}
	HIF1A	0.733787	1.662999	7.11×10^{-40}
	CASP3	-0.33558	0.792465	6.31×10^{-1}
	PPM1L	0.177058	1.130576	3.78×10^{-1}
	ZMYND11	-0.33053	0.795243	4.91×10^{-7}
0-1000	*RARA*	-0.68045	0.623972	1.97×10^{-9}
	FOXC1	1.459043	2.749259	2.25×10^{-1}
	AGO1	-0.10824	0.92772	2.11×10^{-1}
	EID1	0.032808	1.023001	7.21×10^{-1}
	CCND3	-0.12622	0.916231	1.94×10^{-1}
	DNAJB6	-0.05203	0.964576	5.32×10^{-1}
	FERMT2	0.518029	1.431998	6.73×10^{-11}
	RHOC	0.319555	1.247946	4.37×10^{-5}
	ROCK2	-0.12707	0.915688	1.30×10^{-1}
	EIF4EBP1	-0.50253	0.70587	1.32×10^{-9}
	HIF1A	0.739861	1.670015	4.22×10^{-45}
	CASP3	-0.10634	0.928945	8.65×10^{-1}
	PPM1L	0.04847	1.034167	8.49×10^{-1}
	ZMYND11	-0.31568	0.803471	6.01×10^{-7}

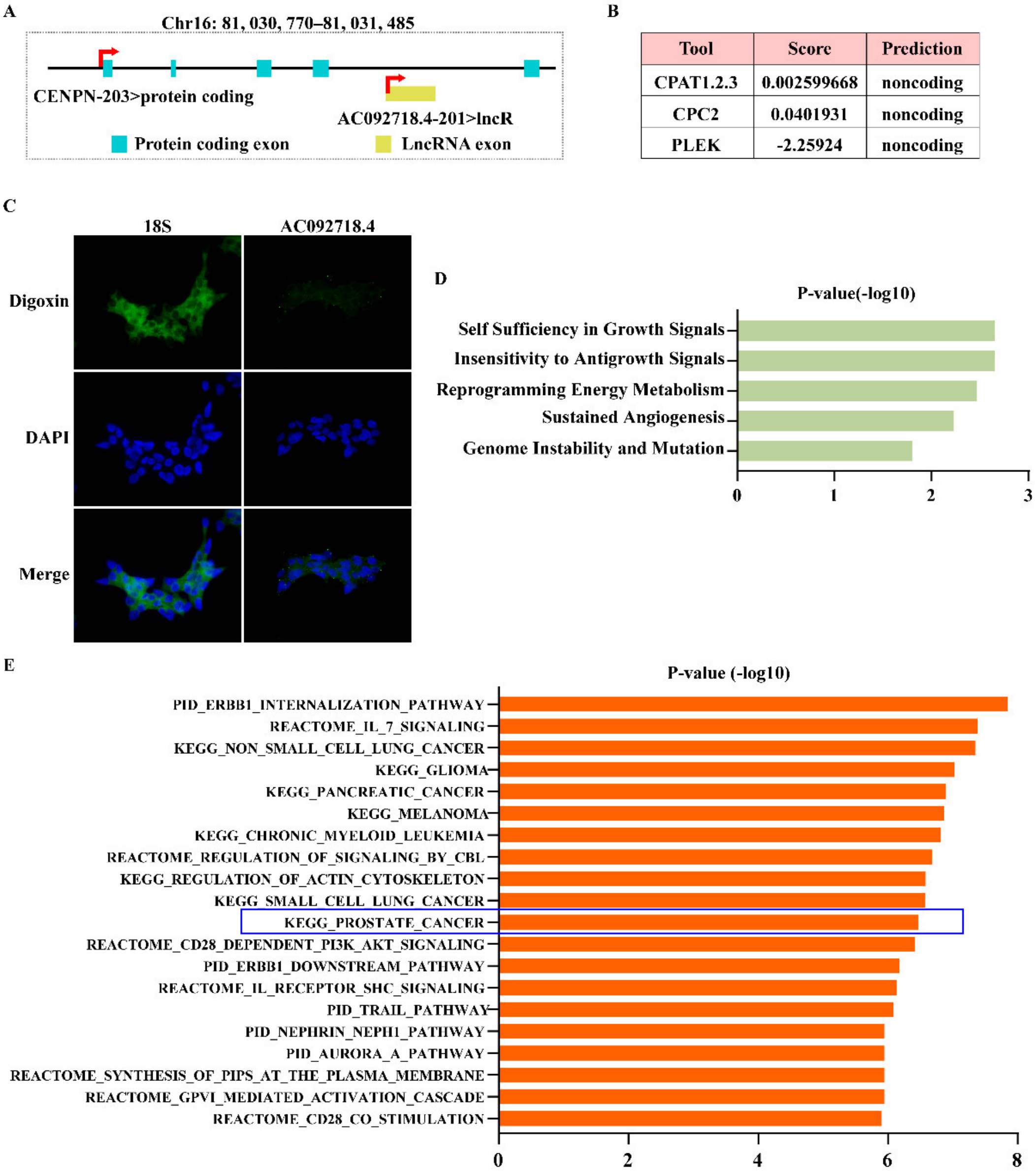

Tool	Score	Prediction
CPAT1.2.3	0.002599668	noncoding
CPC2	0.0401931	noncoding
PLEK	-2.25924	noncoding

Supplementary Figure S1. The molecular characteristic of AC092718.4.

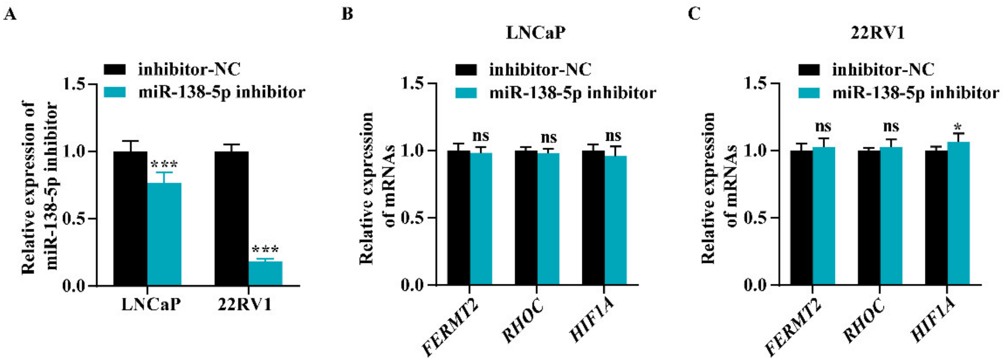

Supplementary Figure S2. The effect of miR-138-5p inhibitor on the expression of target mRNAs.
